# Two and a half decades of United States wildfire burn zone disaster data, 2000-2025

**DOI:** 10.1038/s41597-025-06226-8

**Published:** 2025-12-17

**Authors:** Lauren B. Wilner, Logan Piepmeier, Milo Gordon, Benjamin B. Steiger, Alexander J. Northrop, Heather McBrien, Brittany Shea, Gabriella Y. Meltzer, Neil Singh Bedi, Elizabeth M. Blake, Tarik Benmarhnia, Danielle Braun, Joan A. Casey

**Affiliations:** 1 Department of Epidemiology, University of Washington School of Public Health, Seattle, WA USA; 2 Department of Environmental and Occupational Health Sciences, University of Washington School of Public Health, Seattle, WA USA; 3 Department of Environmental Health Sciences, Columbia University Mailman School of Public Health, New York, NY USA; 4 Department of Pediatrics, The Children’s Hospital of Philadelphia, Philadelphia, Pennsylvania USA; 5 Department of Obstetrics and Gynecology, Columbia University Irving Medical Center, New York, NY USA; 6 Boston University Medical Campus, Boston, MA USA; 7 Harvard T.H. Chan School of Public Health, Boston, MA USA; 8 Scripps Institution of Oceanography, UC San Diego, San Diego, USA; 9 Irset Institut de Recherche en Santé, Environnement et Travail, UMR-S 1085, Inserm, University of Rennes, EHESP, Rennes, France; 10 Department of Data Science, Dana-Farber Cancer Institute, Boston, MA USA

**Keywords:** Environmental impact, Environmental impact, Planetary science

## Abstract

Growing wildfire frequency and urban development expanding into fire-prone areas have heightened wildfire risk for wildland-urban interface (WUI) communities. When burn zones come near or cross into communities, the heat, flames, and smoke can harm human health—directly or via psychosocial stressors—to the point of becoming a disaster. We harmonized six wildfire datasets to create the first U.S.-wide spatial dataset of wildfire burn zone disasters. Our criteria for a wildfire burn zone disaster were wildfires that burned near a community (≥96 people per km^2^ or met WUI criterion) and resulted in ≥1 civilian fatality, ≥1 destroyed structure, or received federal disaster declaration. We identified 6,212 U.S. wildfire burn zone disasters between 2000–2025. The annual number of these disasters ranged between 61 in 2001 and 570 in 2011 (median = 217), with an increasing trend over the study period. California had the highest number of wildfire burn zone disasters (n = 878, 14.1%). These data may inform demographic, economic, and population health research, as well as policymaking and resource allocation.

## Background & Summary

### Increasing risk of wildfire exposure to communities

Wildfires are unplanned, unwanted, or uncontrolled fires that occur in wildlands—highly vegetative regions such as forests, grasslands, or prairies^1^. Wildfires differ from other types of wildland fires that fire management agencies initiate (e.g., prescribed burns) or allow to burn (e.g., wildland fire use events) as part of forest management. Although some types of wildland fires can benefit various species^2–5^, wildfires can harm local ecosystems, agricultural fields^6^, places of cultural significance^7–9^, and human health^10^.

The threat of wildfires to human health is growing, largely due to human activities. Human-caused climate change contributes to larger^11–13^, more frequent^12,14–16^ wildfires and longer wildfire seasons^11,12^. This is in part due to higher ambient temperatures and changes in rainfall that produce drier, more combustible wildland vegetation^15^.

Harmful land use practices can also increase the risk of wildfires. For example, deforestation, the intentional clearing or thinning of forests by humans, has a wide range of ramifications. It reduces wildland humidity because fewer trees remain to release water into the atmosphere (e.g., via transpiration)^17^. Further, deforestation contributes to climate change both directly by reducing available trees to absorb carbon, as well as indirectly. Most deforestation worldwide and in the United States (U.S.) occurs to facilitate carbon-intensive activities and development in the wildland-urban interface (WUI), regions where highly vegetative areas overlap with residences, agriculture, or infrastructure^18–23^. As of 2020, over 44 million homes across the conterminous U.S. were in the WUI^20^, and the number of communities in the WUI is projected to rise. The WUI consists of two types: intermix, where buildings and vegetation directly intermingle, and interface, where buildings are in close proximity to large wildland vegetation areas, but have lower vegetation density than intermix areas^20,23^.

The presence of communities in the WUI can increase the risk of wildfire ignition due to the proximity of combustible materials (e.g., vegetation, buildings, cars)^19,24,25^ to potential human ignition sources. Human ignition sources were responsible for over 80% of wildfires in most regions in the U.S. between 1992 and 2012^26^. Common human ignition sources include power line sparks from outdated electrical systems^27^, fireworks, discarded cigarettes^28^, arson, and other intentional fires that escape human control (e.g., campfires, crop burns, prescribed burns, wildland fire use events). Given the potential for wildfires in the WUI, WUI communities are at high risk of exposure to nearby wildfire burn zones.

### Health risks of wildfire burn zones to nearby communities

Communities near wildfire burn zones may face severe physical and psychosocial threats to health related to their exposure to wildfire smoke and flames. Although wildfire smoke can travel thousands of miles, airborne toxins are likely at higher concentrations closer to the burn zone. Further, wildfires that cross into communities and burn insulation, plastic, vinyl, furniture, upholstery, and other textiles may produce smoke with additional toxic chemicals (e.g., benzene) or heavy metals (e.g., lead) compared to burning vegetation alone^29,30^. Inhalation of wildfire smoke can cause adverse respiratory, cardiovascular, reproductive, and mental health outcomes^10^,^31–41^.

Wildfire smoke can also impact health by disrupting daily life, access to community services, and social networks. Several qualitative studies have found that staying indoors during wildfire smoke events was linked with self-reported stress, anxiety, depression, and feelings of social isolation^34,42,43^.

Sheltering-in-place and evacuating may both carry health risks for nearby residents, however, research is limited and mixed. For example, following the 2018 California Woolsey wildfire, proximity and evacuations were associated with reduced outpatient visits and increased cardiovascular hospitalizations (suggesting disruptions to routine care and increased demand for emergency care, respectively), however no evidence of these changes occurred for the 2019 California Getty wildfire^44^.

When wildfires burn in communities, the damage can be severe. For example, the 2018 California Camp Fire destroyed 18,000 structures—including 14,000 homes—displaced 50,000 people and directly killed 85 people^18^. Wildfire burn damage to homes, businesses, schools, hospitals, power grids, or other infrastructure may displace people from their homes, interrupt education and economic activities, upend social networks, and reduce access to essential services^42,45,46^.

Individuals living near or within wildfire burn zones may experience adverse health effects from directly experiencing or witnessing wildfire-related deaths, injuries, evacuations, healthcare access disruptions, and loss of personal property and community spaces^42^. For example, post-traumatic stress disorder was elevated among evacuees of the 2016 Canadian Fort McMurray wildfire who had witnessed buildings collapse or feared for the safety of a loved one^47^.

### Federal wildfire disaster relief for affected communities

Wildfire suppression, rapid response, and recovery can be costly for nearby communities. Communities near burn zones may face threatened or actual harm, making them eligible for federal wildfire disaster relief from the U.S. Federal Emergency Management Agency (FEMA). To qualify for a Fire Management Assistance Grant (FMAG)—the most common and basic level of wildfire disaster relief from FEMA—a wildfire must threaten lives or improved property, exceed a threshold of state and local firefighting resources, exhibit high fire danger conditions, or threaten major economic impact^48,49^. Between 2016 and 2020, FEMA spent $830 million on 266 FMAGs^49^. This funding provides local/state governments, individuals, and businesses with financial support for wildfire suppression and recovery from wildfire-related losses^49^. Given some of the eligibility criteria for disaster relief (e.g., threats to lives and property), wildfires eligible for disaster relief may overlap with wildfires that burn near and cause harm to nearby communities. However, FEMA’s disaster criteria alone may be insufficient for identifying if a wildfire burned near—and negatively affected—a community.

### Obstacles to defining wildfire burn zone disasters

Although there is no universal set of criteria for defining wildfire disasters (or disasters more generally), most definitions describe disasters as events that harm communities. However, organizations and researchers may use different criteria to measure harm and, thus, may disagree on when a wildfire constitutes a disaster. For example, some environmental and health groups recognize wildfire smoke events as disasters^50^. Kumagai and colleagues (2004) argued that a wildfire disaster “…enters a human settlement, burns people’s homes and businesses, and disrupts their lives^51^.” Their definition captures many of the potential harmful effects of wildfires with burn zones near or overlapping communities; however, it neglects other potential measures of community harm.

**Fig. 1:**
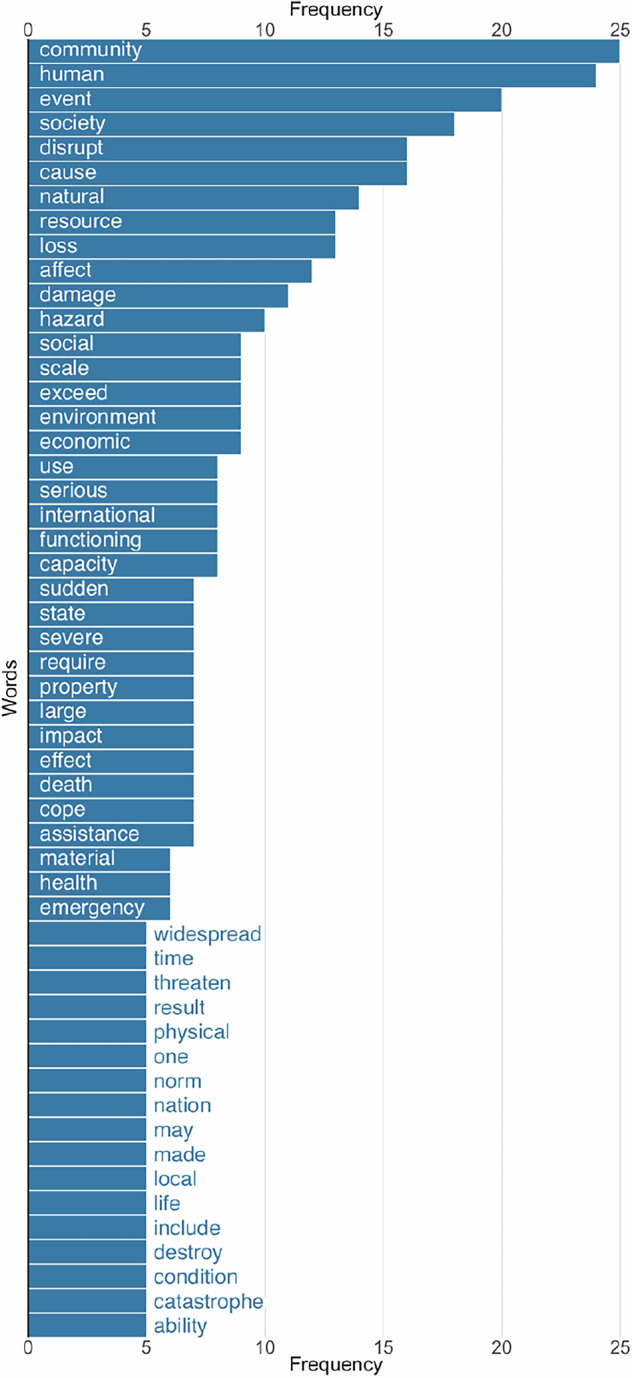
Bar plot of words associated with 31 disaster definitions.

Other disaster definitions include themes of harm to human health; damage to the natural and built environment; economic losses; disruption of community services; the need for external (e.g., federal or international) aid; and collective trauma from directly experiencing or witnessing threats to the safety of oneself or others (Fig. 1)^52–59^.

Clearly defined disaster criteria may assist researchers in identifying wildfire burn zones that harm nearby communities. Criteria that use widely available data may allow researchers to construct large-scale wildfire burn zone disaster datasets.

### Obstacles to creating a wildfire burn zone disaster dataset

The current literature on health effects of nearby wildfires focuses on single wildfire events. Most wildfire health studies identify study populations using data on wildfire evacuees^47,60^, wildfire relief recipients^61^, wildfire burn injury patients^62^, and individuals living, working, or attending a school or hospital in the county or census-designated place where a wildfire occurred^45,63,64^.

Large-scale, multi-wildfire, multi-state epidemiologic studies on wildfire burn zone disasters would offer greater generalizability about associations between wildfire burn zone disasters and health outcomes. Such research could inform targeted public health strategies to prepare for and respond to wildfire burn zone disasters. However, to our knowledge, no such studies on wildfire burn zone disasters exist^65^, likely due to data limitations. Although various local, state, and federal agencies produce wildfire datasets in the U.S., there is no national comprehensive, standardized system for reporting data on wildfires. By extension, there are no national datasets on wildfire burn zone disasters.

Harmonizing existing wildfire datasets presents challenges because agencies often use different (and sometimes internally inconsistent) practices to identify wildfires. In addition, datasets span different time periods and geographic regions and adhere to agency-dependent reporting guidelines^66^.

Groups that have harmonized wildfire datasets have used various methods and inclusion criteria. For example, federal and state agencies at the National Interagency Fire Center (NIFC) created a national spatial dataset of wildfire burn zones using data from eight state and federal government agencies^66^. The U.S. Geological Survey (USGS) created a national dataset by harmonizing data from 40 government agencies^67^. Although both datasets contain useful spatial data on wildfire burn zones, they do not include data on community harm.

Although FEMA disaster relief declaration data are publicly available^68^ and may indicate threatened or actual harm to communities, FEMA’s dataset does not record specific information on community harm (e.g., civilian fatalities, evacuations) or the distance between burn zones and communities. Further, the FEMA dataset only included wildfires that met the criteria for federal funding, which may exclude some wildfire burn zone disasters.

St. Denis et al. previously used the U.S. Incident Command System Form 209 (ICS-209) to create the comprehensive ICS-209-PLUS wildfire dataset^69^. This dataset spanned 1999–2020 and contained nationwide information on wildfire-related fatalities, structural damage, and burn zone spatial extents^70,71^. The ICS-209-PLUS dataset contains additional hazards beyond wildfires, including hurricanes, floods, tornadoes, and winter storms, and has the stated objective of enabling “exploration of the daily progression of the most costly, damaging, and deadly environmental-hazard events^69^.” Our dataset is complementary to ICS-209-PLUS in several ways. First, we added CalFIRE Redbooks data, enhancing our dataset for California fires and providing disaggregated total and civilian fatality counts by wildfire. Second, we incorporated indicators of FMAG declarations. Third, we linked population density data and WUI data to identify community harm and provide a measure of wildfire severity. Fourth, we determined whether the wildfire burn zone disaster intersects with intermix, interface WUI, or neither type. The most recent iteration of the ICS-209-PLUS dataset links to two spatial datasets–Monitoring Trends in Burn Severity (MTBS) and Fire Events Delineation (FIRED). We additionally link to NIFC. By differentiating between wildfire burn zone disasters from other types of wildfires, we endeavored to provide researchers with a dataset to assess the effects of wildfires that burn near or within communities.

### Our aim: create a wildfire burn zone disaster dataset

We aimed to build on existing burn zone datasets by first defining a wildfire burn zone disaster and then harmonizing existing datasets with information on community harm and FMAG declarations. In this paper, we created a spatial dataset of U.S. wildfire burn zone disasters from 2000–2025 using the following steps:

1. We defined wildfire burn zone disasters using four criteria: civilian fatalities, destroyed structures, federal assistance, and proximity to communities.
2. We identified datasets with information relevant to our criteria.
3. We cleaned U.S. wildfire datasets with data on our disaster criteria and filtered them to 2000–2025.
4. We combined datasets with data on fatalities, structural damage, and FMAGs.
5. We filtered to wildfires with at least one fatality, destroyed structure, or FMAG.
6. We linked burn zone spatial data from existing datasets.
7. We identified wildfires with burn zones near or intersecting communities.

Our final dataset provided each wildfire burn zone disaster’s ignition and containment dates, spatial extent (where available), burned area, counts of total fatalities, civilian fatalities, destroyed or damaged structures, whether the wildfire received an FMAG declaration, and whether it was near a community, among other factors. Such a dataset may have utility for demographic, economic, and population health research, as well as for policymaking and resource allocation.

## Methods

We created a spatial dataset of U.S. wildfire burn zone disasters from 2000 to June 15, 2025. First, we conducted a literature review to identify and select metrics for defining a wildfire burn zone disaster. Then, we harmonized publicly available wildfire datasets to create a complete dataset of U.S. wildfire burn zones and identified wildfires that met our disaster criteria. After data cleaning, we determined whether these wildfires affected communities to produce our final wildfire burn zone disaster dataset.

### Defining wildfire burn zone disasters

To define a wildfire burn zone disaster, we conducted a literature review of disaster definitions and then selected disaster criteria corresponding to enough existing data to create a multi-decade wildfire burn zone disaster dataset spanning the entire U.S.

For the literature review, we included peer-reviewed manuscripts and government and non-governmental organization websites. Previous reviews on disasters indicated a lack of consensus on a disaster definition^72^, which we similarly found when comparing definitions. We used 31 disaster definitions from researchers, government, and non-governmental groups and identified common themes (Fig. 1)^52,58,73–99^. A major theme of disaster definitions was their effect on communities as opposed to individuals. Other recurring themes included disruptions to community functioning, the need for external assistance, damage to infrastructure or the environment, and harm to human health (e.g., death, injury, collective trauma).

Based on these disaster themes, we identified corresponding metrics in existing U.S. wildfire datasets to define a wildfire burn zone disaster (Table 1). We selected metrics that were widely available across the U.S. and were likely to be measured in the future.

**Table 1:** Data availability of wildfire burn zones disaster effects.

Type of disaster effect	Examples of effects	Feasible metric	Dataset availability
Human health	Death, injuries, collective trauma, threats to safety or health	Number of deaths	ICS-209; Redbooks
Material	Damage or destruction of property, infrastructure, or critical resources; economic losses	Number of destroyed structures	ICS-209; Redbooks
Political	Exceeds ability of a community to cope or recover; governmental disaster declarations; governmental request for external aid; require emergency response; require external support for recovery	Received an FMAG declaration	FMAG dataset
Social	Disruption to the functioning of a community; broken social ties	Wildfire intersects a community	Wildfire spatial datasets, GHSL population data, and SILVIS WUI data

FEMA, Federal Emergency Management Agency; FMAG, Fire Management Assistance Grant; GHSL, Global Human Settlement Layer; ICS-209, Incident Command System Form 209. SILVIS WUI, Spatial Analysis For Conservation and Sustainability Lab Wildland-Urban Interface.

First, we considered harm to human health using data on fatalities. To meet our fatality criterion, a wildfire needed to result in at least one civilian fatality. If the fatality breakdown (i.e., by firefighter versus civilian) was unavailable, we required a wildfire to cause at least one fatality of any type.

Second, we considered harm to the physical environment using data on affected structures (e.g., homes or commercial properties). To meet our structure criterion, a wildfire needed to result in at least one destroyed structure or (if destroyed structure data was unavailable) at least one damaged/destroyed structure.

Third, we identified the use of external assistance using data on FMAGs. A wildfire needed to receive an FMAG to meet our FMAG criteria.

Finally, to assess community harm (as opposed to individual harm), we identified which wildfire burn zones were near or overlapping a community. We defined a community in two ways. First, we used a minimum population density threshold of ≥250 people per square mile (≥96 people per square kilometer), which is the minimum population density of an interface WUI community according to U.S. fire management agencies^46,100^. The optimal distance threshold for defining wildfire exposure in health, well-being, and economic studies is unknown. Prior health research has classified individuals as exposed if they lived within 15–20-km of wildfire burn zones^44,101^. Because larger burn zones may pose risks to communities further away, we determined that wildfires met our community proximity criterion if the burn zone was within 10-km of a community for small wildfires (<1,000 acres) and within 20-km for large wildfires (≥1,000 acres). Second, we used Spatial Analysis For Conservation and Sustainability Lab (SILVIS) Global Wildland-Urban Interface Dataset, which classifies areas based on building density, to determine which wildfire burn zones overlapped with intermix or interface WUI communities^23^. We considered a wildfire to affect a community if it met either our population density or SILVIS WUI criterion.

A wildfire constituted a wildfire burn zone disaster if it met any of our three criteria for human harm (at least one civilian fatality, destroyed structure, or FMAG declaration) and overlapped or was near a community. Our final dataset contains additional variables that allow researchers to define the level of intensity or severity of the wildfire burn zone disaster, including the ignition and containment dates (duration), average and maximum population density in the buffered wildfire burn zone, WUI classification, burn zone area, threatened structures, number of injuries, number of people evacuated, and estimated cost.

### Identifying datasets with data relevant to wildfire burn zone disasters

#### Wildfire burn zone disaster criteria datasets

We obtained information on our wildfire burn zone disaster criteria for human harm from two national and one California-specific sources. We provide data dictionaries for each criterion dataset in our documentation hosted on GitHub (https://github.com/lpiep/wfbz_disasters_lite) and Harvard Dataverse^102^.

##### ICS-209 Large Incident Summaries Datasets

The U.S. Department of Agriculture and the U.S. Department of the Interior jointly operate https://wildfire.gov, which makes available annual database backups of all historical Incident Status Summary reports (form ICS-209) for 1999 through 2024 (archived at the end of each year). For more recent form data, we acquired access to the U.S. National Wildfire Coordinating Group’s data API^103^.

This ICS-209 database included information on wildfire-related fatalities and destroyed structures, which we used as disaster criteria, plus additional related variables such as civilian and responder injuries, evacuations, threatened structures, and forecasted response cost.

##### FMAG dataset

FEMA produces a Disaster Declarations Summary dataset, which contains all official Federal Disaster Declarations from 1953 to the present (June 2025) for all-hazard events (e.g., hurricanes, wildfire incidents)^68^. The dataset included a variable to indicate the type of disaster declaration (e.g., major disaster, emergency, or FMAG). We filtered to FMAG declarations.

The FMAG dataset included the disaster’s title and whether several FEMA programs were declared (i.e., Individuals and Households, Individual Assistance, Public Assistance, and Hazard Mitigation). The dataset did not contain data on civilian fatalities, destroyed structures, or proximity to communities, providing only information on one of our disaster criteria—receipt of an FMAG declaration. Unlike the ICS-209 dataset, the FMAG dataset did not provide the size of the burn zone, point of origin coordinates, or any linking ID variables to spatial datasets.

The FMAG dataset did contain information for identifying wildfires, including an incident ID variable, incident/complex name, time variables (year, ignition date, declaration date, and containment date), and location variables (state, county, and designated area).

##### Redbooks dataset

The California Department of Forestry & Fire Prevention (CAL FIRE) publishes annual reports on wildfires called Redbooks^104^. The 2000–2008 Redbooks were not originally publicly available. We acquired PDFs of all Redbooks between 2000–2008 and CSVs of all Redbooks between 2008–2023 directly from CAL FIRE, which we have made available on GitHub: https://github.com/lpiep/wfbz_disasters_lite/tree/main/data/01_raw/event/redbook.

Each annual Redbooks report included a data table for “large fires.” Between 2000–2007, Redbooks defined large fires as wildfires that burned ≥30 acres of timber, ≥300 acres of brush, ≥1500 acres of woodland, ≥1500 acres of grass, ≥1500 acres of agricultural products, destroyed ≥ three structures, or caused ≥$300,000 in damage. Between 2008–2023, Redbooks defined large fires as wildfires that burned ≥300 acres, regardless of type of vegetation burned.

The Redbooks large fire tables (herein referred to as Redbooks) contained data on the same two disaster criteria as ICS-209: civilian fatalities and destroyed structures. Redbooks reported civilian fatalities for all study years. However, from 2007–2009, the Redbooks combined the counts of damaged and destroyed structures into a single variable. Before 2007 and after 2009, Redbooks included two distinct variables for damaged and destroyed structures. Redbooks did not include proximity to communities, however they provided burn zone size (acres burned). Unlike ICS-209, Redbooks did not contain point of origin coordinates or linking ID variables to spatial datasets.

Other Redbooks variables included the incident/complex name, time variables (year, county, ignition date, containment date), and cause.

#### Spatial wildfire datasets

We were unable to find spatial wildfire datasets already containing any of our disaster criteria. However, we found five national spatial datasets containing wildfire burn zone spatial extents, which we use to identify wildfires with burn zones near or overlapping communities. We used three datasets: MTBS, FIRED, and NIFC. We excluded two datasets because they are no longer maintained and do not span our entire study period: The USGS Geospatial Multi-Agency Coordination Group (GEOMAC)^105^ published data until 2020 when its data and ongoing responsibilities were shifted to NIFC; and Welty and Jeffries at the USGS published a dataset harmonizing historical fires through 2020^67^.

The wildfire burn zone datasets used different inclusion criteria, contributing data sources, spatial and temporal resolutions, and methodologies, resulting in distinct burn zone end products. Both MTBS and FIRED derived burn zone perimeters from a land use change algorithm, while NIFC relied on numerous input data. We provide data dictionaries for each spatial dataset in our documentation hosted on GitHub (https://github.com/lpiep/wfbz_disasters_lite/tree/main/docs-site/docs/raw_data_desc).

##### MTBS dataset

MTBS is a federal interagency program that provides spatial datasets for large fires across the contiguous U.S., Alaska, Hawaii, and Puerto Rico from 1984 to 2024^106^.

All MTBS burn zone data through 2015 came from National Aeronautics and Space Administration (NASA) Landsat satellite imagery. Beginning in 2015, MTBS incorporated data from the European Space Agency Sentinel-2 satellite program, which provided additional observations and increased the availability of optimal imagery for mapping wildfire burn zones. Analysts used pre- and post-fire scenes to create Normalized Burn Ratio (NBR), differenced Normalized Burn Ratio (dNBR), and Relativized differenced Normalized Burn Ratio (RdNBR) images. From these images along with reflectance imagery, analysts digitized perimeters that included any detectable burned areas for each wildfire. MTBS used the NAD83 projection (EPSG: 4269) and a 30-meter resolution.

The MTBS dataset linked to the ICS-209 dataset via *Integrated Reporting of Wildland Fire Information* identifier (IRWIN ID) wherever possible, and name/location/ignition date where IRWIN IDs were unavailable. Additional variables included incident/complex name, time variables (year of ignition, ignition date), state, acres burned, and a categorical variable that distinguished the type of wildland fire (e.g., wildfire, prescribed fire, wildland fire use event, unknown cause).

A drawback of MTBS was that it only included large fires, which MTBS defined as ≥ 1000 acres in the Western U.S. and ≥ 500 in the Eastern U.S. MTBS defined the Western U.S. as states west of Minnesota, Iowa, Missouri, Arkansas, and Louisiana, including Alaska and Hawaii.

##### FIRED dataset

The FIRED dataset included a GeoPackage containing the estimated total burn zones of wildfires in the contiguous U.S. and Alaska from November 2001 to July 2024^107,108^. The FIRED dataset relied on sub-daily, 500-meter resolution raster grids of wildfire-burned areas. These grids were produced from satellite imagery from the NASA Moderate Resolution Imaging Spectroradiometer (MODIS) Collection 6 MCD64 Burned Area product^107^. The FIRED dataset uses a flexible spatiotemporal window to identify wildfire events^108^ and the MODIS sinusoidal projection.

The FIRED dataset linked to the ICS-209 dataset via point of origin and date. Additional variables in FIRED included ignition date, containment date, and acres burned.

The FIRED dataset contained over three times as many wildfires as MTBS by including smaller wildfires. The FIRED dataset lacked 8,721 wildfires from the MTBS dataset, likely due to MODIS’ inability to detect wildfires in the presence of clouds, smoke, or intact canopy, and because MTBS relies on reports of large fires to guide the mapping process^109^. Unlike the MTBS dataset, the FIRED dataset lacked an incident name or ID variable.

##### NIFC dataset

Federal and state agencies at NIFC produced a dataset of wildfires and prescribed burns across the contiguous U.S., Alaska, and Hawaii from 2000-present (June 2025)^110^. The NIFC dataset included data from eight federal and state agencies. These individual agencies used different methodologies to develop wildfire burn zone perimeters, including ground-based incident reporting, human observation, administrative records, and remotely sensed data. The NIFC dataset used a WGS84 geographic coordinate reference system (EPSG: 4326).

Variables included an ID variable, year of wildfire ignition, state, burned acres, comments, and burn zone geometry. NIFC was linked to ICS-209 via IRWIN ID wherever possible, and name/location/ignition date where IRWIN IDs were unavailable.

A drawback of the NIFC dataset was that it lacked an ignition date variable.

#### Non-wildfire datasets

We used census county and state shapefiles and a city-county dataset to assist in cleaning wildfire location data. We also used a population raster dataset and a WUI dataset to assess the community proximity criterion.

##### Census datasets

We downloaded 2000, 2010, and 2020 U.S. census state and county shapefiles from the Census TIGER database^111^. The shapefiles used the NAD83 USA Contiguous Albers Equal Area Conic projection (ESPG: 102003). We used these shapefiles to identify counties affected by burn zones in the spatial wildfire datasets, clean county name variables from the non-spatial wildfire datasets and harmonize the wildfire datasets.

##### SimpleMaps city-county dataset

We downloaded a dataset from SimpleMaps^112^ that included the names of cities or towns and their corresponding counties and states. We used this dataset to clean county name variables from the non-spatial wildfire datasets.

##### Global human settlement layer dataset

The Global Human Settlement Layer (GHSL) provided spatial raster datasets of population at 100 m^2^ resolution in 5-year intervals^113,114^. We downloaded GHSL population grids for 2000, 2005, 2010, 2015, and 2020. We assigned each wildfire a GHSL population grid by rounding wildfire’s ignition year to the nearest 5-year interval and matching it to the corresponding GHSL population grid. We used this dataset to estimate population density and generate our community intersection criterion.

##### SILVIS global wildland-urban interface dataset

The University of Wisconsin SILVIS Lab used remote sensing datasets of vegetation and building footprints to classify land along several wildland-urban interface and intermix categories at a high resolution globally^23^. We selected this dataset over the U.S. Forest Service’s WUI dataset because the SILVIS global dataset included Alaska, Hawaii, and Caribbean U.S. territories.

The SILVIS dataset classifies land by land cover (forest/shrub/wetland, grassland, urban, or other) and WUI (interface, intermix, or non-WUI) at a 10-meter resolution. We classified each burn zone perimeter according to whether any part of it intersected with intermix WUI areas, interface WUI areas, both, or neither. Intermix WUI communities were defined as blocks where wildland vegetation covered >50% of the area and that had at least 6.17 houses/km^2^ (>1 house per 40 acres). Interface WUI communities were defined as blocks where wildland vegetation covered <50% of the area and that were located within 2.4 km of an area at 5 km^2^ in size with ≥75% wildland vegetation cover. These WUI variables were added to the final dataset and used as one of the two criteria to identify whether wildfire burn zones overlapped a community.

### Cleaning wildfire datasets

#### Cleaning event names

Existing wildfire datasets differed in their content and conventions for identifying wildfire incidents. We cleaned all variables by standardizing their formats. We standardized all fire names according to USPS standard abbreviations (e.g., “Mountain” to “MTN”)^115^ and standardized county names. We also removed data erroneously included in the fire datasets, such as COVID-19 disaster declarations found in the ICS-209 data.

#### Cleaning event dates

For all datasets, we excluded any events from prior to 2000 based on the ignition year. All the datasets included ignition date variables except for NIFC, which only contained ignition year. We cleaned and standardized all date variables: ignition, containment, and FMAG Declaration dates.

#### Converting Redbooks PDFs to CSVs

Although we obtained CSVs for annual Redbook large fire tables between 2008 and 2023, all Redbook reports between 2000 and 2007 were only available as PDFs. We converted the PDFs to CSVs using the ABBYY FineReader PDF program and kept rows and columns containing data from the Redbooks large fire tables. We then combined all Redbooks large fire tables into a 2000–2023 Redbook dataset.

#### Harmonizing and cleaning ICS-209 data

The ICS-209 database schema changed over time, tracking different form entries in different variables, so we created a harmonized dataset of selected variables (available at https://github.com/lpiep/ics209_minimal). The identifying variables included in the ICS-209 datasets were incident name, location (both state/county and the coordinates of the point of origin), and ignition date. Some historical data schemas also included the IRWIN ID, which allowed linkage to the MTBS and NIFC spatial datasets.

The raw variables from which we obtained disaster criteria variables (e.g., wildfire-related fatalities and destroyed structures) changed according to the historical data schema. A table defining the source and availability of these variables in each schema is available at the GitHub repository above.

#### Cleaning administrative location identifiers in event datasets

We used several methods to determine the names of the wildfire-affected states and counties (or county equivalents, referred to collectively as “counties” hereafter) depending upon the dataset and available variables. We standardized all county names to match the names found in the U.S. Census TIGER dataset and wrote code to detect the frequently abbreviated but important Los Angeles County. When multiple counties were included in the datasets, we retained all of them, and when multiple states and counties were provided, we retained all combinations of state and county name. We used these state and county names to link datasets when shared IRWIN ID or point of origin were unavailable.

Of the 35,961 wildfire events identified from 2000–2025 from the harmonized ICS-209 dataset, 32,981 (91.7%) had county names provided and 35,093 (97.6%) had latitude and longitude of the point of origin provided. All the 1,126 events identified from the Redbooks dataset had a county name provided. We assumed all events in the Redbooks datasets were in California. Of the 1,731 FEMA FMAG declarations identified for our time period of interest, 1,693 (97.8%) had county names provided.

#### Cleaning spatial datasets

For the three spatial wildfire datasets, we removed invalid and multi-surface geometries and recast all polygon topologies into multi-polygons. We identified affected states and counties by intersecting the wildfire burn zones with county boundaries from U.S. Census TIGER database. We transformed all spatial data to NAD83 (EPSG 4269).

### Harmonizing datasets with fatality, structure, and FMAG data

**Fig. 2:**
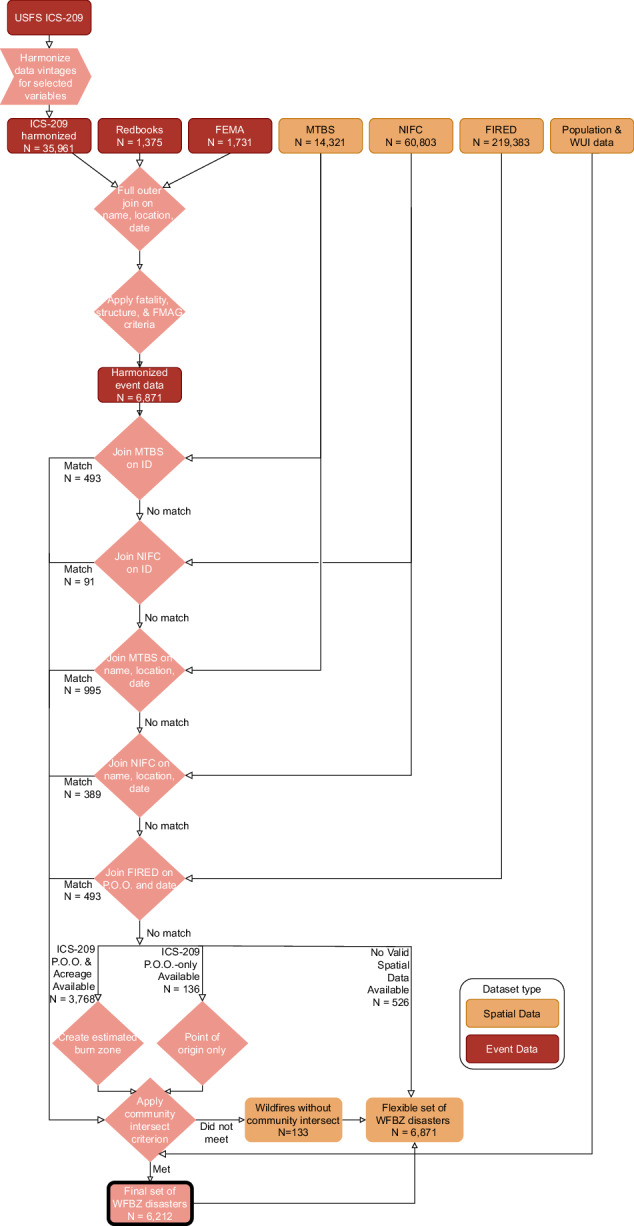
Flow diagram detailing the construction of a 2000–2025 U.S. wildfire burn zone disaster dataset. FEMA, Federal Emergency Management Agency; FIRED, Fire Event Delineation; FMAG, Fire Management Assistance Grant; ICS-209, Incident Command System Form 209; NIFC, National Interagency Fire Center; MTBS, Monitoring Trends in Burned Severity; P.O.O., point of origin; USFS, U.S. Forest Service; WFBZ, wildfire burn zone; WUI, wildland-urban interface.

We next harmonized the ICS-209, Redbooks, and FMAG datasets (Fig. 2) by identifying matching wildfires. We matched wildfires based on ignition date ($\le$30 days apart), incident/complex names, cleaned county name, and states. We used fuzzy matching when incident/complex names or county names did not exactly match. Fires were considered to match if their recorded county or counties overlapped, the ignition dates were within 30 days of each other, and they shared similar names (calculated using the “stringdist” R package^116^ and defined as a Jaro-Winkler string distance of less than or equal to 0.25 with a prefix factor of 0.1).

When the wildfires were part of a complex, we aggregated individual fires to complexes. When we identified a fire or complex that appeared in multiple datasets but where the datasets disagreed on a name for the fire or complex after standardizing, we retained all the names given for that event.

Both the ICS-209 and Redbooks datasets contained information on fatalities and destroyed structures. When these two datasets conflicted, we used the value from Redbooks. The ICS-209, Redbooks, and FMAG datasets all contained ignition and containment dates. When these conflicted, we again used the value from Redbooks, and if Redbooks did not contain the event, we used the earliest dates in ICS-209 or FMAG.

### Selecting wildfires that met the fatality, structure, or FMAG criteria

Next, we used variables from the ICS-209, Redbooks, and FMAG datasets to filter for wildfires that met the fatality, structure, or FMAG disaster criteria.

Some years in ICS-209 and Redbooks were missing either civilian fatality data (2000–2013 for ICS-209) or destroyed structure data (2007–2009 for Redbooks). Therefore, wildfires met our fatality criterion if they resulted in at least one reported civilian fatality or—for wildfires between 2000–2013—if ICS-209 reported at least one fatality of any type. Wildfires met our destroyed structure criterion if they resulted in at least one reported destroyed structure or—for wildfires between 2007 and 2009—if Redbooks reported at least one damaged structure.

Wildfires met the FMAG criterion if they appeared in the FMAG dataset, the sole source of FMAG data.

Wildfires that met one or more of these criteria comprised the *preliminary wildfire burn zone disaster dataset*. To identify which wildfires met our community proximity criterion, we assessed population densities within and around wildfire burn zones, as well as WUI located within the burn zone. We included wildfire burn zones that met our three criteria (fatality, destroyed structure, or FMAG) in our final dataset, regardless of whether they met our community proximity criterion, allowing future data users to apply alternative population density and WUI thresholds as needed.

### Providing wildfire burn zone spatial data

When possible, we linked wildfires from our preliminary wildfire burn zone disaster dataset to one of the three wildfire burn zone spatial files using the best available identifying information (Fig. 2).

We first attempted to link to MTBS and FIRED by IRWIN IDs, which were available for some ICS-209 wildfires. When IRWIN IDs were unavailable, we matched to MTBS by name, location, and ignition date. Next, we matched to NIFC using wildfire names, location, and ignition year (no ignition date available).

We allowed some flexibility in spatial and temporal matching. When matching on counties, we included county matches when the wildfire was within 10 km of a county. When matching on ignition date, we allowed matches within 30 days of any listed ignition date (or 30 days before or after the year listed in NIFC, since only the year of the fire was available).

After matching our preliminary wildfire burn zone data set to the spatial datasets, we merged any remaining disjoint linkages. For example, if fire *A* had been linked to fire *B*, and fire *B* had been linked to fire *C*, but fire *C* had not been linked to fire *A*, we merged all three wildfires into a single observation. In these cases, we retained the geometry according to the same order of spatial data precedence as above, retained the maximum number of structures destroyed and fatalities, and assigned an FMAG declaration if any of the fires in the group had an FMAG. We retained the minimum ignition date, FMAG data, and the maximum containment date.

Using MTBS, FIRED, and NIFC, we matched 2,461 of our preliminary wildfire burn zone disasters to wildfire spatial perimeters (Fig. 2).

For the 4,430 wildfires without a match to a wildfire spatial perimeter, 3,768 wildfires had feasible point of origin coordinates (latitude/longitude) and data on burn zone area from the ICS-209 dataset (Fig. 2). For each of these wildfires, we approximated the burn zone by creating a circular buffer using the reported burn zone size as the circle’s area and the reported point of origin as the circle’s center.

Of the remaining wildfires, 136 had a feasible point of origin from ICS-209 but lacked data on burn zone size; we retained these wildfires in the dataset as point geometries (Fig. 2). An additional 526 wildfires were kept in the final dataset, but had no spatial data beyond reported county or state.

### Determining which wildfires met the community proximity criterion

We evaluated which wildfire burn zones overlapped or were near communities (regions with a population density of ≥96 people per km^2^)^46,100^ by assessing the highest population density of regions within or surrounding each burn zone.

The GHSL population grids contained population estimates at a 100-m^2^ resolution across the US^113,114^. We calculated the population density within each grid cell by dividing the number of people by 100 m^2^. We assigned each grid cell the weighted average of population density of all grids within a 300-m radius, creating a smooth population density data layer at a 100-m^2^ resolution.

Next, we created spatial buffers around the wildfire burn zones. For small wildfire burn zones (<1,000 acres or burn zone with only point of origin), we created 10-km buffers around the burn zone. For larger wildfire burn zones (≥1,000 acres), we created 20-km buffers.

We then intersected the smooth population density raster with the wildfire burn zone buffers to identify the maximum population density grid cell value for each wildfire burn zone. If the maximum population density value was ≥96 people per km^2^, we flagged the wildfire as meeting our population density criteria. We retained the maximum population density for all grid cells within the buffered burn zone. For each wildfire burn zone, we also determined and retained the average population density among all grid cells within the buffered burn zone.

Next, we performed a spatial join with the SILVIS WUI dataset to provide an additional measure of whether a wildfire burn zone overlapped a community. The SILVIS WUI dataset classifies WUI intermix based on building density rather than population density. When fires overlapped with either an area reaching the population density threshold of 96 people per km^2^ or with the WUI intermix or interface, we flagged that wildfire event as occurring in a community.

### Ongoing data updates for wildfire burn zone disasters, 2025-onward

The code, environment, documentation, and dataset are publicly available on Harvard Dataverse^102^ and GitHub and we aim to update the data annually to include additional years as long as input data streams and analyst time remain available. Herein, we describe the dataset posted to Harvard Dataverse in September 2025.

## Data Records

The 2000–2025 wildfire burn zone disaster dataset contains a list of the cleaned wildfire incident and complex names (all variations are included). A disaster ID variable uniquely identifies each wildfire/wildfire complex, and additional ID variables link to the raw source datasets. Temporal variables include the ignition date (earliest and latest), containment date (earliest and latest), and FMAG declaration date. Geographic data describing the burn zone include state, county, point of origin coordinates, burn zone area in km^2^, and burn zone geometries with geometry source and the method used to link that geometry to the criteria dataset.

The dataset contains variables characterizing the wildfire burn zone disaster criteria. Disaster variables include the number of civilian fatalities, total fatalities, destroyed structures, FMAG declarations, and whether the wildfire met the community proximity criterion.

Additionally, we provide variables for average and maximum population density in the buffered wildfire burn zone, WUI classification, threatened structures, number of injuries, number of people evacuated, and estimated cost.

**Fig. 3:**
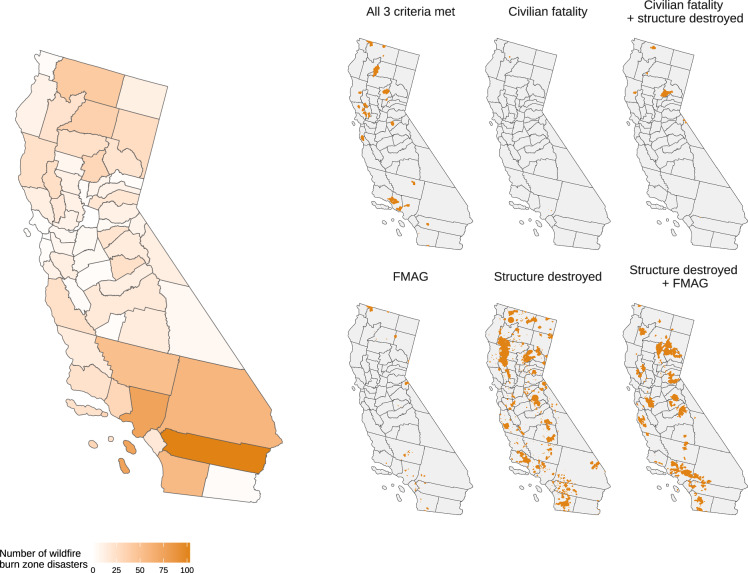
Disaster criteria met by California wildfire burn zone disasters between 2000–2025. FMAG, Fire Management Assistance Grant from FEMA.

The dataset includes 6,871 wildfires that resulted in at least one civilian fatality, at least one destroyed structure, or an FMAG declaration. Of these wildfires, 6,212 (90.4%) met the community proximity criterion and qualified as wildfire burn zone disasters (Figs. 2, 3).

Among the 6,212 wildfire burn zone disasters, we provided 2,392 with burn zones using wildfire spatial perimeters, 3,697 with burn zone approximations using circular buffers, and 123 with points of origin only (Fig. 2).

Between 2000–2025, the majority of wildfire burn zone disasters met the structure criterion (n = 5,717 [92.0%]) compared to 930 (15.0%) that met the FMAG criterion and 101 (1.6%) that met the fatality criterion (Fig. 2). In total, the wildfire burn zone disasters resulted in 120,634 destroyed structures and 407 civilian/total fatalities between 2000–2025.

**Fig. 4:**
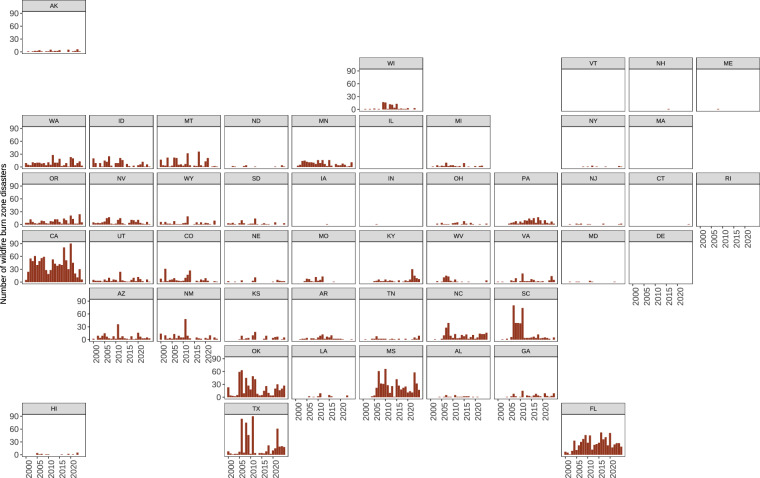
The number of wildfire burn zone disasters by state from 2000–2025. Wildfire burn zone disasters were near or overlapping communities and resulted in at least one destroyed structure, civilian fatality, or FMAG declaration. Wildfire burn zone disasters that overlapped with multiple states appear in each corresponding state panel. Note: The y-axis is truncated at 90 incidents for visual clarity. Texas experienced 130 wildfire burn zone disasters in 2011, which extended beyond the displayed range. FMAG, Fire Management Assistance Grant from FEMA.

The annual number of wildfire burn zone disasters fluctuated from 61 in 2001 to 570 in 2011 (Fig. 4). We observed an increasing trend in wildfire burn zone disasters over the 26-year period (Mann-Kendall τ = 0.997, *p* < 0.001). The number of wildfire burn zone disasters increased from 2,350 (2000–2009) to 2,630 (2010–2019), with corresponding increases in disasters meeting specific criteria: fatalities (13 to 52), structures (2,192 to 2,360), and FMAG (319 to 373). The states with the greatest number of annual wildfire burn zone disasters each year were California (2002–2004, 2012–2014, 2017, 2019–2021), Florida (2001, 2009, 2016, 2018), Mississippi (2010, 2015, 2019, 2023–2024), Oklahoma (2005), South Carolina (2007), and Texas (2000, 2006, 2008–2009, 2011, 2022) (Fig. 4). These six states accounted for 54% of all wildfire burn zone disasters in our dataset.

From 2000–2025, California had the most wildfire burn zone disasters (n = 878, (14.1%)), total civilian fatalities (n = 213, [52.3%]), destroyed structures (n = 854,417, [70.0%]), and wildfires with FMAGs (n = 228, [24.5%]).

Most wildfire burn zone disasters resulted in no fatalities and destroyed few structures. Among the 101 wildfire burn zone disasters that met the fatality criterion, 77.2% resulted in ≤ 2 fatalities. Among the 5,982 wildfire burn zone disasters that met the structure criterion, 4,611 (80.7%) destroyed ≤ 4 structures. Only 636 (10.2%) wildfire burn zone disasters resulted in >10 fatalities or >10 destroyed structures. However, these 636 wildfires accounted for 344 (84.5%) civilian fatalities and 110,274 (91.4%) destroyed structures. The 2018 California Camp Fire alone was responsible for 85 (20.6%) fatalities and 18,804 (14.7%) destroyed structures.

Wildfire burn zone disasters affected areas with a median (IQR) maximum population density of 2700 (1163, 6809) people per km^2^. 3,298 (53.1%) disasters overlapped with intermix WUI alone, 73 (1.2%) overlapped with the interface WUI alone, and 593 (9.6%) overlapped with both WUI types. Just 327 (5.2%) of wildfires had any reported evacuations. Among wildfires with any people evacuated, a median of 210 (IQR: 50, 1,175) were evacuated. Among the 2,261 (36.4%) of wildfires with estimated cost of wildfire response, the median cost was $37,000 (IQR: $1,200, $2,000,000).

## Technical Validation

We used several strategies to validate our code and data at all steps of development.

### Automated data pipeline

We used an automated data pipeline tool, the R Targets package^117^, to run the data pipeline and automatically produce data quality metrics. This pipeline tool ensures that the output dataset was always consistent with the code and any source data and that basic quality checks were run every time the data were reproduced.

### Manual quality checks

We iteratively inspected the output dataset and improved processing code, creating flexible rules that produced valid data. Examples of manual quality checks we performed include checking for the presence of known fires in the data, mapping the burn zones to check that they had reasonable locations and sizes, and checking that missingness was reasonable and distributed consistently over time and space.

### Comparison to ICS-209-PLUS

The ICS-209-PLUS dataset has the goal to track progression of cost, damage, and fatalities of U.S. wildfires and other incidents. Our dataset builds on previous wildfire datasets, including the comprehensive ICS-209-PLUS dataset, by incorporating additional variables and data sources. We designed our dataset with the end goal of facilitating demographic, economic, and population health research, as well as policymaking and resource allocation.

We compared our nationwide wildfire burn zone disaster dataset to the existing ICS-209-PLUS dataset for the years of overlap (2000–2020) and for fires we could match on the incident ID from ICS-209-PLUS. For this comparison, we did not require our wildfire burn zone disasters to meet the community overlap criterion, as we could not apply it in the ICS-209-PLUS dataset. We matched 4,984 (82.5% of fires) from our dataset to ICS-209-PLUS using the ICS ID variable. Of these matched fires, 46 lacked spatial data within the ICS-209-PLUS dataset.

We assessed differences in the two datasets in terms of number of wildfire burn zone disasters, number of structures destroyed, number of civilian and total fatalities, burn area (km^2^), and ignition dates.

**Fig. 5:**
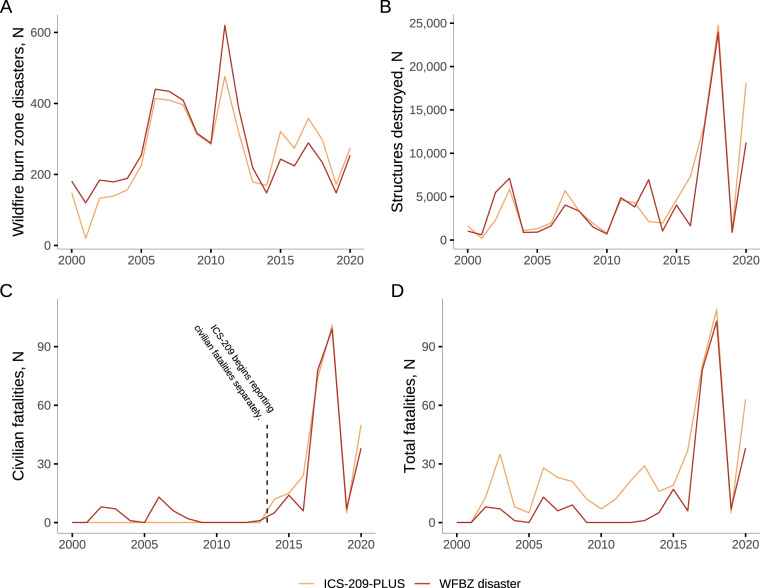
Comparison of our final U.S. wildfire burn zone disaster dataset versus ICS-209-PLUS (2000–2020) by (**A**) Wildfire burn zone disasters, (**B**) Destroyed structures, (**C**) Civilian fatalities, and (**D**) Total fatalities. ICS-209, Incident Command System Form 209; WFBZ, wildfire burn zone.

Prior to 2014, we identified slightly more annual total wildfire burn zone disasters than the ICS-209-PLUS dataset (Fig. 5A), and ICS-209-PLUS identified more from 2014-2020. The median (IQR) difference in annual total disasters between the two datasets was 33 (24, 64). We generally observed similar counts of destroyed structures each year comparing our dataset to ICS-209-PLUS (median difference = 472, IQR = 293, 1273; Fig. 5B). In 2020, the destroyed structure counts differed significantly, when we estimated 11,239 and ICS-209-PLUS 18,098 destroyed structures. We also saw similar counts of civilian fatalities between our dataset and ICS-209-PLUS after 2013 when ICS-209 began reporting civilian fatalities separately (2014–2020 median difference = 4, IQR = 2, 10; Fig. 5C). Larger differences emerged when comparing total fatalities between the two datasets (median difference = 11, IQR = 5, 17; Fig. 5D).

**Fig. 6:**
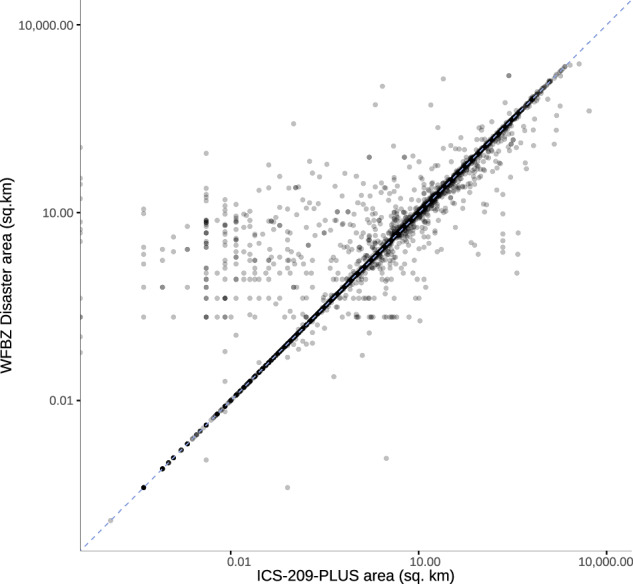
Correlation between burn zone area (km^2^) for wildfires in our final U.S. wildfire burn zone disaster dataset and ICS-209-PLUS (2000–2020). ICS-209, Incident Command System Form 209; WFBZ, wildfire burn zone.

The two datasets showed a strong correlation in reported burn area (Pearson *r* = 0.85, Spearman $\rho$ = 0.94, Fig. 6), and a median (IQR) difference of 0.86 km^2^ (0.02, 10.5).

Ignition date matched between the two data sources 97.7% of the time, with a mean difference of 0.3 days (SD = 3.8).

## Data Availability

The data described in this article are publicly available at the Harvard Dataverse repository (10.7910/DVN/DWILBW)^118^. The dataset includes the following files: • **wfbz_disasters_2000**–**2025.geojson**: A GeoJSON file containing spatial data for 6,212 wildfire burn zone disasters across the U.S. from 2000 to 2025. This harmonized dataset identifies wildfires that burned near communities and resulted in one or more civilian fatalities, one or more destroyed structures, or federal disaster relief. The file also includes an additional 153 wildfire burn zones that did not intersect with a community and 506 wildfire burn zones that lacked spatial data beyond state and county level, but that met the fatality, structure, or Fire Management Assistance Grant (FMAG) criteria. **• wfbz_disasters_data_dictionary_2000**–**2025.md**: A data dictionary file providing detailed variable definitions, data collection methods, and metadata. We built the national wildfire burn zone disaster dataset by harmonizing six wildfire datasets. The annual number of disasters in the dataset ranges from 61 (in 2001) to 570 (in 2011), with a median of 217 disasters per year. Data are made available under a CC0 1.0 Universal Public Domain Dedication, allowing for unrestricted use. The dataset will be kept current and updated annually.

## Code availability

Code is available on GitHub: https://github.com/lpiep/wfbz_disasters_lite. We performed all data cleaning, combining of datasets, and summary statistics in R Version 4.4.2, primarily using *dplyr, stringr, lubridate*, and *sf*. Population density criterion checks were performed using Python Version 3.13.1, primarily using *pandas* and *GeoPandas*.

## References

[1] NWCG Glossary of Wildland Fire Terminology. National Wildfire Coordinating Group. https://www.nwcg.gov/publications/pms205/nwcg-glossary-of-wildland-fire-pms-205 Accessed July 9 2022.

[2] Pausas, J. G. & Keeley, J. E. Wildfires as an ecosystem service. *Frontiers in Ecology and the Environment.* **17**(5), 289–295 (2019).

[3] van Wagtendonk, J. W. The History and Evolution of Wildland Fire Use. *Fire Ecology.* **3**(2), 3–17 (2007).

[4] Doane, D. L., O’Laughlin, J. Morgan, P. & Miller, C. Barriers to wildland fire use: A preliminary problem analysis. *International Journal of Wilderness*. **12**(1), 36–38 (2006).

[5] What is a Prescribed Fire? National Park Service. https://www.nps.gov/articles/what-is-a-prescribed-fire.htm. Accessed July 10 2022.

[6] Kabeshita, L. *et al*. Pathways framework identifies wildfire impacts on agriculture. *Nature Food.* **4**(8), 664–672 (2023). 37550540 10.1038/s43016-023-00803-z

[7] LaPier, R. R. Analysis: Burning of Lahaina’s sacred sites is a major loss for Native Hawaiians. But their history will live on. *PBS News Hour*. https://www.pbs.org/newshour/nation/analysis-burning-of-lahainas-sacred-sites-is-a-major-loss-for-native-hawaiians-but-their-history-will-live-on. Accessed July 11 2024.

[8] Peters, J. First Nations lament cultural losses to B.C. wildfires. *CBC News*. https://www.cbc.ca/news/canada/british-columbia/first-nations-wildfires-cultural-losses-1.6945729. Accessed July 1 2023.

[9] Kelleher, J. S. & Hong, J. C. Maui fires renew centuries-old tensions over water rights. The streams are sacred to Hawaiians. *AP News*. https://apnews.com/article/hawaii-maui-fires-water-streams-531263684bf5106d635f29aec91115e4. Accessed July 20 2025.

[10] Gould, C. F. *et al*. Health Effects of Wildfire Smoke Exposure. *Annu Rev Med*. **75**, 277–292 (2024). 10.1146/annurev-med-052422-020909 PMC12183787 37738508

[11] Wildfire climate connection. National Oceanic and Atmospheric Administration. https://www.noaa.gov/noaa-wildfire/wildfire-climate-connection. Accessed July 11 2023.

[12] Climate Change Indicators: Wildfires. U.S.Environmental Protection Agency. https://www.epa.gov/climate-indicators/climate-change-indicators-wildfires#ref4. Accessed July 11 2023.

[13] Dennison, P. E., Brewer, S. C., Arnold, J. D. & Moritz, M. A. Large wildfire trends in the western United States, 1984–2011. *Geophysical Research Letters.* **41**(8), 2928–2933 (2014).

[14] Abatzoglou, J. T. & Williams, A. P. Impact of anthropogenic climate change on wildfire across western U.S.forests. *Proc Natl Acad Sci USA* **113**(42), 11770–11775 (2016). 27791053 10.1073/pnas.1607171113 PMC5081637

[15] Zhuang, Y., Fu, R., Santer, B. D., Dickinson, R. E. & Hall, A. Quantifying contributions of natural variability and anthropogenic forcings on increased fire weather risk over the western United States. *Proc Natl Acad Sci USA* **118**(45) (2021). 10.1073/pnas.2111875118 PMC8609294 34725162

[16] Wehner, M. F., Arnold, J. R., Knutson, T., Kunkel, K. E. & LeGrande, A. N. Droughts, floods, and wildfires. In: Wuebbles DJ, D. W. Fahey, K. A. Hibbard, D. J., Dokken, B. C. Stewart, and T. K. Maycock, ed. *Climate Science Special Report: Fourth National Climate Assessment*. Vol I. Washington, DC, USA: U.S. Global Change Research Program; (2017).

[17] Feng, X. *et al*. How deregulation, drought and increasing fire impact Amazonian biodiversity. *Nature.* **597**(7877), 516–521 (2021). 34471291 10.1038/s41586-021-03876-7

[18] Butler, K. *et al*. Wildland-Urban Interface (WUI) Fire Data Collection on Parcel Vulnerabilities. https://www.nist.gov/programs-projects/wildland-urban-interface-wui-fire-data-collection-parcel-vulnerabilities. Accessed November 21 2023.

[19] Radeloff, V. C. *et al*. Rapid growth of theU.S.wildland-urban interface raises wildfire risk. *Proc Natl Acad Sci USA* **115**(13), 3314–3319 (2018). 29531054 10.1073/pnas.1718850115 PMC5879688

[20] Radeloff, V. C. *et al*. The 1990-2020 wildland-urban interface of the conterminous United States-geospatial data. *USDA Research Data Archive* (2022).

[21] Angelsen, A. & Kaimowitz, D. Rethinking the causes of deforestation: Lessons from economic models. *World Bank Res Obser.* **14**(1), 73–98 (1999). 10.1093/wbro/14.1.73 12322119

[22] Deforestation: causes and how the EU is tackling it. European Parliament. https://www.europarl.europa.eu/topics/en/article/20221019STO44561/deforestation-causes-and-how-the-eu-is-tackling-it. Accessed August 6 2025.

[23] Schug, F. *et al*. The global wildland-urban interface. *Nature.* **621**(7977), 94–99 (2023). 37468636 10.1038/s41586-023-06320-0 PMC10482693

[24] Leber, R. How power companies have caused and worsened wildfires. *Vox*. https://www.vox.com/science-and-health/2022/9/13/23345164/california-wildfires-heat-blackouts-burning. Accessed December 1 2022.

[25] Penn, I. & Eavis, P. Hawaiian Electric Was Warned of Its System’s Fragility Before Wildfire. *New York Times*. https://www.nytimes.com/2023/08/19/business/energy-environment/hawaiian-electric-maui-wildfire-climate-change.html. Accessed Aug 19 2023.

[26] Balch, J. K. *et al*. Human-started wildfires expand the fire niche across the United States. *Proc Natl Acad Sci USA* **114**(11), 2946–2951 (2017). 28242690 10.1073/pnas.1617394114 PMC5358354

[27] Brown, M., Fassett, C., Whittle, P., McConnaughey, J. & Lo, J. Storms batter aging power grid as climate disasters spread. *AP News*. https://apnews.com/article/wildfires-storms-science-business-health-7a0fb8c998c1d56759989dda62292379. Accessed April 5 2022.

[28] Short, K. C. A spatial database of wildfires in the United States, 1992-2011. *Earth Syst Sci Data.* **6**(1), 1–27 (2014).

[29] National Academies of Sciences, Engineering, and Medicine. The Chemistry of Fires at the Wildland-Urban Interface. *The National Academies Press.*10.17226/26460 (2022). 36657007

[30] Krasovich Southworth, E. *et al*. The Influence of Wildfire Smoke on Ambient PM(2.5) Chemical Species Concentrations in the Contiguous US. *Environ Sci Technol*. **59**(6), 2961–73 (2025). 10.1021/acs.est.4c09011 39899563

[31] Navarro, K. M. *et al*. Wildland firefighter smoke exposure and risk of lung cancer and cardiovascular disease mortality. *Environ Res.* **173**, 462–468 (2019). 30981117 10.1016/j.envres.2019.03.060

[32] Neumann, J. E. *et al*. Estimating PM2.5-related premature mortality and morbidity associated with future wildfire emissions in the western US. *Environ Res Lett*. **16**(3) (2021). 10.1088/1748-9326/abe82b PMC8048092 33868453

[33] Cascio, W. E. Wildland fire smoke and human health. *Sci Total Environ.* **624**, 586–595 (2018). 29272827 10.1016/j.scitotenv.2017.12.086 PMC6697173

[34] Eisenman, D. P. & Galway, L. P. The mental health and well-being effects of wildfire smoke: a scoping review. *BMC Public Health.* **22**(1), 2274 (2022). 36471306 10.1186/s12889-022-14662-z PMC9724257

[35] Reid, C. E. & Maestas, M. M. Wildfire smoke exposure under climate change: impact on respiratory health of affected communities. *Curr Opin Pulm Med.* **25**(2), 179–187 (2019). 30461534 10.1097/MCP.0000000000000552 PMC6743728

[36] Chen, H., Samet, J. M., Bromberg, P. A. & Tong, H. Cardiovascular health impacts of wildfire smoke exposure. *Part Fibre Toxicol.* **18**(1), 2 (2021). 33413506 10.1186/s12989-020-00394-8 PMC7791832

[37] Reid, C. E. *et al*. Critical Review of Health Impacts of Wildfire Smoke Exposure. *Environ Health Perspect.* **124**(9), 1334–1343 (2016). 27082891 10.1289/ehp.1409277 PMC5010409

[38] Holm, S. M., Miller, M. D. & Balmes, J. R. Health effects of wildfire smoke in children and public health tools: a narrative review. *Journal of Exposure Science & Environmental Epidemiology.* **31**(1), 1–20 (2021). 32952154 10.1038/s41370-020-00267-4 PMC7502220

[39] Elser, H. *et al*. Wildfire Smoke Exposure and Incident Dementia. *JAMA Neurol.* **82**(1), 40–48 (2025). 39585704 10.1001/jamaneurol.2024.4058 PMC11589856

[40] Heft-Neal, S. *et al*. Emergency department visits respond nonlinearly to wildfire smoke. *Proc Natl Acad Sci USA* **120**(39), e2302409120 (2023). 37722035 10.1073/pnas.2302409120 PMC10523589

[41] Ma, Y. *et al*. Long-term exposure to wildland fire smoke PM2. 5 and mortality in the contiguous United States. *Proc Natl Acad Sci USA* **121**(40), e2403960121 (2024). 39316057 10.1073/pnas.2403960121 PMC11459178

[42] Humphreys, A., Walker, E. G., Bratman, G. N. & Errett, N. A. What can we do when the smoke rolls in? An exploratory qualitative analysis of the impacts of rural wildfire smoke on mental health and wellbeing, and opportunities for adaptation. *BMC Public Health.* **22**(1), 41 (2022). 34991532 10.1186/s12889-021-12411-2 PMC8740038

[43] Mottershead, K. D., McGee, T. K. & Christianson, A. Evacuating a First Nation Due to Wildfire Smoke: The Case of Dene Tha’ First Nation. *International Journal of Disaster Risk Science*. **11**(3), 274–286 (2020).

[44] McBrien, H. *et al*. Wildfire Exposure and Health Care Use Among People Who Use Durable Medical Equipment in Southern California. *Epidemiology.* **34**(5), 700–711 (2023). 37255240 10.1097/EDE.0000000000001634 PMC10524711

[45] Hamideh, S., Sen, P. & Fischer, E. Wildfire impacts on education and healthcare: Paradise, California, after the Camp Fire. *Natural Hazards.* **111**(1), 353–387 (2022). 34658527 10.1007/s11069-021-05057-1 PMC8500817

[46] Schulze, S. S., Fischer, E. C., Hamideh, S. & Mahmoud, H. Wildfire impacts on schools and hospitals following the 2018 California Camp Fire. *Nat Hazards.* **104**, 901–925 (2020).

[47] Belleville, G. *et al*. Psychological symptoms among evacuees from the 2016 Fort McMurray wildfires: a population-based survey one year later. *Frontiers in Public Health*. **9**, 655357 (2021). 34017813 10.3389/fpubh.2021.655357 PMC8130827

[48] Fire Management Assistance Grant Program and Policy Guide. FEMA. https://www.fema.gov/sites/default/files/documents/fema_fmagppg_063121.pdf. Accessed August 10 2024.

[49] Congressional Budget Office. Wildfires. https://www.cbo.gov/publication/58212. Accessed July 10 2022.

[50] Lim, M., Riddle, K. & Pfeiffer, S. Petition pushes FEMA to classify extreme heat and wildfire smoke as ‘major disasters’. https://www.npr.org/2024/06/18/nx-s1-5010416/petition-pushes-fema-to-classify-extreme-heat-and-wildfire-smoke-as-major-disasters. Accessed July 1 2025.

[51] Kumagai, Y., Carroll, M. S. & Cohn, P. Coping with Interface Wildfire as a Human Event: Lessons from the Disaster/Hazards Literature. *Journal of Forestry.* **102**(6), 28–32 (2004).

[52] McFarlane, A. C. & Norris, F. H. Definitions and Concepts in Disaster Research. In Methods for Disaster Mental Health Research, edited by F. H. Norris, S. Galea, M. J. Friedman, and P. J. Watson. Guilford Press, 2006.

[53] Barnett, J., Dennis-Rouse, M. & Martinez, V. Wildfire disaster leads to facilities evacuation. *Orthop Nurs.* **28**(1), 17–20 (2009). 19190472 10.1097/01.NOR.0000345849.32424.0a

[54] Karmarkar, E. *et al*. Outbreak of Norovirus Illness Among Wildfire Evacuation Shelter Populations - Butte and Glenn Counties, California, November 2018. *MMWR Morb Mortal Wkly Rep.* **69**(20), 613–617 (2020). 32437337 10.15585/mmwr.mm6920a1 PMC7357343

[55] McCaffrey, S., Wilson, R., & Konar, A. Should, I Stay or Should I Go Now? Or Should I Wait and See? Influences on Wildfire Evacuation Decisions. *Risk Anal.* **38**(7), 1390–1404 (2018). 29168989 10.1111/risa.12944

[56] Hurteau, M. D., Westerling, A. L., Wiedinmyer, C. & Bryant, B. P. Projected effects of climate and development on California wildfire emissions through 2100. *Environ Sci Technol.* **48**(4), 2298–2304 (2014). 24443984 10.1021/es4050133

[57] Afolabi, M. O. Public Health Disasters: A Global Ethical Framework. 12, 1–24 (2018).

[58] What is a disaster? International Federation of the Red Cross. https://www.ifrc.org/our-work/disasters-climate-and-crises/what-disaster. Accessed July 11 2023.

[59] Thériault, L., Belleville, G., Ouellet, M. C. & Morin, C. M. The Experience and Perceived Consequences of the 2016 Fort McMurray Fires and Evacuation. *Front Public Health.* **9**, 641151 (2021). 34858911 10.3389/fpubh.2021.641151 PMC8632018

[60] Verstraeten, B. S. E., Elgbeili, G., Hyde, A., King, S. & Olson, D. M. Maternal Mental Health after a Wildfire: Effects of Social Support in the Fort McMurray Wood Buffalo Study. *Can J Psychiatry.* **66**(8), 710–718 (2021). 33172310 10.1177/0706743720970859 PMC8320544

[61] Marshall, G. N., Schell, T. L., Elliott, M. N., Rayburn, N. R. & Jaycox, L. H. Psychiatric disorders among adults seeking emergency disaster assistance after a wildland-urban interface fire. *Psychiatr Serv.* **58**(4), 509–514 (2007). 17412853 10.1176/ps.2007.58.4.509

[62] Wasiak, J. *et al*. 12-month generic health status and psychological distress outcomes following an Australian natural disaster experience: 2009 Black Saturday Wildfires. *Injury.* **44**(11), 1443–1447 (2013). 23021367 10.1016/j.injury.2012.08.060

[63] Agyapong, B. *et al*. Mental Health Impacts of Wildfire, Flooding and COVID-19 on Fort McMurray School Board Staff and Other Employees: A Comparative Study. *Int J Environ Res Public Health*. **19**(1) (2021). 10.3390/ijerph19010435 PMC8744856 35010692

[64] Brown, M. R. G. *et al*. Significant PTSD and Other Mental Health Effects Present 18 Months After the Fort Mcmurray Wildfire: Findings From 3,070 Grades 7-12 Students. *Front Psychiatry.* **10**, 623 (2019). 31543839 10.3389/fpsyt.2019.00623 PMC6728415

[65] Grant, E. & Runkle, J. D. Long-term health effects of wildfire exposure: A scoping review. *The Journal of Climate Change and Health.* **6**, 100110 (2022).

[66] National Interagency Fire Center. (2022). InterAgencyFirePerimeterHistory All Years View [Data set]. https://data-nifc.opendata.arcgis.com/datasets/nifc::interagencyfireperimeterhistory-all-years-view/about

[67] Welty, J. & Jeffries, M. Combined wildland fire datasets for the United States and certain territories, 1800s-Present:U.S.Geological Survey data release.U.S. *Geological Survey*. (2021).

[68] Federal Emergency Management Agency. OpenFEMA Dataset: Disaster Declarations Summaries - v2. https://www.fema.gov/openfema-data-page/disaster-declarations-summaries-v2. Accessed August 5 2025.

[69] St. Denis, L. A. *et al*. All-hazards dataset mined from the U.S. National Incident Management System 1999–2020. *Scientific Data.* **10**(1), 112 (2023). 36828905 10.1038/s41597-023-01955-0 PMC9958120

[70] St. Denis, L. A., Mietkiewicz, N. P., Short, K. C., Buckland, M. & Balch, J. K. All-hazards dataset mined from the U.S.National Incident Management System 1999–2014. *Scientific Data.* **7**(1), 64 (2020). 32081906 10.1038/s41597-020-0403-0 PMC7035274

[71] Short, K. C. Spatial wildfire occurrence data for the United States, 1992-2020 [FPA_FOD_20221014]. In: Archive FSRD, ed. 6th edition ed. Fort Collins, CO. 2022.

[72] Mayner, L. A. P. Defining disaster: The need for harmonisation of terminology. *Australasian Journal of Disaster and Trauma Studies.* **19**, 21–25 (2015).

[73] Resilience, AIfD. *Disaster Health: Handbook 1*. Australia (2011).

[74] Bibby, D. J. *Disaster Dictionary: The Definitive Guide to Related Terms, Acronyms and Concepts for Emergency Planning and Operations* 1ed: CRC Press; (2005).

[75] Turner, B. A. *Man-made disasters*. London: Wykeham Publications; (1978).

[76] Iowa Legislature. Chapter 29C: Emergency Management and Security. https://www.legis.iowa.gov/DOCS/ACO/IC/LINC/Chapter.29C.html. Accessed August 10 2025.

[77] Gender and Health in Disasters. In: Health DoGaWs, ed. 20, Avenue Appia, Geneva Switzerland: World Health Organization; (2002).

[78] Thywissen, K. *Components of Risk. A Comparative Glossary*. Bonn, Germany (2006).

[79] *World Disasters Report: A Focus on Public Health*. Dordrecht, The Netherlands: International Federation of Red Cross and Red Crescent Societies; (2000).

[80] *Australian Disaster Resilience Handbook 1: Disaster Health*. Australian Institute for Disaster Resilience CC BY-NC; (2011).

[81] Dynes, R. R. Coming to terms with community disaster. In: Quarantelli E. L., ed. *What is a Disaster? Perspectives on the Question*. 109–126 (New York: Routledge; 1998).

[82] Noji, E. K. The public health consequences of disasters. *Prehosp Disaster Med.* **15**(4), 147–157 (2000). 11227602

[83] Fritz, C. E. Disaster. In: Merton, R.K. and Nisbet, R.A., Eds., Contemporary Social Problems, Harcourt, Brace and World, New York, 651–694 (1961).

[84] Glossary of bioterrorism and public health emergency terms and acronyms. Washington State Department of Health. https://www.ncbi.nlm.nih.gov/books/NBK98393/. Accessed August 20 2025.

[85] Report of the open-ended intergovernmental expert working group on indicators and terminology relating to disaster risk reduction. In: Assembly U. N. G., ed: United Nations General Assembly. https://www.preventionweb.net/files/50683_oiewgreportenglish.pdf. Accessed August 1 2024.

[86] Disaster. United Nations Office for Disaster Risk Reduction (UNDRR). The Sendai Framework Terminology on Disaster Risk Reduction Web site. https://www.undrr.org/terminology/disaster. Accessed 5 March (2025).

[87] Sundnes, K. O. & Birnbaum, M. L. Health Disaster Management: Guidelines for Evaluation and Research in the Utstein Style. *Prehospital and Disaster Medicine*. **17**, 144–161 (2003). 10558317

[88] O’Leary, M. R. *The Dictionary of Homeland Security and Defense*. Lincoln, NE: iUniverse, Inc.; (2006).

[89] State and Local Guide 101: Guide for All-Hazard Emergency Operations Planning. In: FEMA, ed (1996).

[90] Biby, D. J. *Disaster Dictionary. The Definitive Guide to Related Terms, Acronyms, and Concepts for Emergency Planning and Operations*. Tulsa OK, USA: K & M Publishers Inc.; (2005).

[91] GÖ, B. Mental Health Consequences of COVID-19 Disaster. *Infectious Diseases & Clinical Microbiology*. **2020**(1), 42–45.

[92] Natural Disasters. Department of Homeland Security. https://www.dhs.gov/archive/natural-disasters. Accessed Dec 17 2024.

[93] IPCC. *Managing the Risks of Extreme Events and Disasters to Advance Climate Change Adaptation. A Special Report of Working Groups I and II of the Intergovernmental Panel on Climate Change*. Cambridge University Press, Cambridge, UK, and New York, NY, USA (2012).

[94] Guha-Sapir, D., Hoyois, P. & Below, R. *Annual Disaster Statistical Review 2015: The numbers and trends*. Brussels, Belgium: Centre for Research on the Epidemiology of Disasters Institute of Health and Society (2016).

[95] Mayner, L. & Arbon, P. Defining disaster: The need for harmonisation of terminology. *Australasian Journal of Disaster and Trauma Studies*. 19(Special Issue) (2015).

[96] Disaster Management and Public Health. Athar Institute of Health & Management Studies. https://aihms.in/blog/disaster-management-and-public-health/. Accessed July 10 2025.

[97] Sjoberg, D., Whiting, K., Curry, M., Lavery, J. & Larmarange, J. Reproducible summary tables with the gtsummary paakage. *The R Journal.* **13**, 570–580 (2021).

[98] Factsheet: care of the dead in disasters. In: *World Health Organization Regional Office for the Western Pacific*. https://www.wpro.who.int/media_centre/fact_sheets/fs_20061207.htm. Accessed September 1 2025.

[99] Glossary of Terms. Centre for Disaster Protection. https://www.disasterprotection.org/glossary. Accessed July 20 2022.

[100] U.S. Department of Agriculture and U.S. Department of the Interior. Urban Wildland Interface Communities within the Vicinity of Federal Lands That Are at High Risk from Wildfire. Federal Register 66, 751-777, https://www.federalregister.gov/documents/2001/01/04/01-52/urban-wildland-interface-communities-within-the-vicinity-of-federal-lands-that-are-at-high-risk-from (2001)

[101] Johnston, D., Önder, Y., Rahman, M. & Ulubasoglu, M. Evaluating wildfire exposure: Using wellbeing data to estimate and value the impacts of wildfire. *Journal of Economic Behavior & Organization.* **192**, 782–798 (2021).

[102] Piepmeier, L., Casey, J. A. & Wilner, L. B. United States wildfire burn zone disaster data, 2000 to 2025. In. *V3 ed: Harvard Dataverse*; (2025). 10.1038/s41597-025-06226-8 PMC12712048 41408113

[103] U.S. Department of Agriculture and U.S. Department of the Interior. Wildland Fire Application Information Portal. https://www.wildfire.gov/application/sit209. Accessed May 12 2025.

[104] Cal Fire. (2009-2020). Redbooks [Data set]. https://www.fire.ca.gov/our-impact/statistics.

[105] U.S. Geological Survey. Where can I find wildfire perimeter data? https://www.usgs.gov/faqs/where-can-i-find-wildfire-perimeter-data. Accessed September 5 2025.

[106] U.S. Geological Survey and U.S Department of Agriculture. Monitoring Trends in Burn Severity. https://www.mtbs.gov/. Accessed July 11 2024.

[107] Mahood, A. L. *et al*. FIRED CONUS-AK [Data set]. https://scholar.colorado.edu/concern/datasets/d504rm74m.

[108] St. Balch, J. K. *et al*. FIRED (Fire Events Delineation): An Open, Flexible Algorithm and Database of U.S.Fire Events Derived from the MODIS Burned Area Product (2001–2019). *Remote Sensing.* **12**(21), 3498 (2020).

[109] Short, K. C. Sources and implications of bias and uncertainty in a century ofU.S.wildfire activity data. *International Journal of Wildland Fire.* **24**(7), 883–891 (2015).

[110] National Interagency Fire Center. InterAgencyFirePerimeterHistory All Years https://data-nifc.opendata.arcgis.com/datasets/nifc::interagencyfireperimeterhistory-all-years-view/about. Accessed June 3 2025.

[111] U.S. Census Bureau. TIGER/Line Shapefiles. https://www.census.gov/geographies/mapping-files/time-series/geo/tiger-line-file.html. Accessed April 3 2025.

[112] SimpleMaps. Data Made Simple. https://simplemaps.com/. Accessed September 20 2024.

[113] Freire, S., MacManus, K., Pesaresi, M., Doxsey-Whitfield, E. & Mills, J. Development of new open and free multi-temporal global population grids at 250 m resolution. *Population.* **250**, 33 (2016).

[114] Schiavina, M. F. S., Carioli, A. & MacManus, K. GHS-POP R2023A - GHS population grid multitemporal (1975-2030). European Commission, Joint Research Centre (JRC). http://data.europa.eu/89h/2ff68a52-5b5b-4a22-8f40-c41da8332cfe. Accessed June 12 2024.

[115] U.S. Postal Service. Mailing Standards of the United States Postal Service Publication 28 - Postal Addressing Standards. https://pe.usps.com/text/pub28/welcome.htm. Accessed April 3 2025.

[116] van der Loo, M. P. J. The stringdist package for approximate string matching. The R Journal, 6(1), 111-122. https://CRAN.R-project.org/package=stringdist. Accessed Jul 17 2025.

[117] Landau, W. The targets R package: a dynamic Make-like function-oriented pipeline toolkit for reproducibility and high-performance computing. *J Open Source Softw.* **6**(57), 2959 (2021).

[118] Casey, J. *et al*. Two and a half decades of United States wildfire burn zone disaster data, 2000-2025. *Harvard Dataverse.*10.7910/DVN/DWILBW (2025). 10.1038/s41597-025-06226-8 PMC12712048 41408113

